# Supplementary material for: Citizenship status and career self-efficacy: An intersectional study of biomedical trainees in the United States
Source: PLoS One. 2024 Mar 20;19(3):e0296246. doi: 10.1371/journal.pone.0296246 (PMC10954142; doi:10.1371/journal.pone.0296246)
Supplement: S1 File — S1 Table. Summary of study variables, key constructs, common abbreviations, & survey questions. S2 Table. Inter-reliability of Items. S3 Table. Full model ANOVA: 4-WAY ANOVA (Self-Efficacy with Gender x Citizenship x PI Career x Seniority). S4 Table. Contrasts (with Tukey corrections). S5 Figures. Additional Career Pathway Figures. (PDF) [file pone.0296246.s001.pdf]

S1 Table. Summary of study variables, key constructs, common abbreviations, & survey questions

Variable name	Transformed	Survey Question(s) Used	Scale	Notes & Abbreviations
Career Self-Efficacy	Bivariate	Assess your abilities to pursue your desired career path(s)<br>Determine the steps to pursue your desired career path(s)<br>Seek advice from professionals in your desired career path(s)<br>Identify potential employers, firms, and institutions relevant to your desired career path(s)<br>Achieve your career goals	1=Not at all confident<br>2=Minimally confident<br>3=Moderately confident<br>4=Highly confident<br>5=Completely confident	SCCT=Social Cognitive Career Theory<br><br>CSE= Career Self-Efficacy
Seniority	Bivariate	Years in current position	Junior vs. Senior	Graduate Student: Junior = 3rd year and below; Senior = 4th Year and up<br><br>Postdoc: Junior = 1st year and below; Senior = 2nd year and up
Gender	N/A	What is your gender? (optional)	1=Male<br>2=Female	
PI Career Interest	Bivariate	To what extent are you currently considering [the career path of] Principal investigator in a research-intensive institution	1=Not at all considering<br>2=Slightly considering<br>3=Moderately considering<br>4=Strongly considering<br>5=Will definitely pursue	PI=Principal Investigator (research-intensive)<br>a) If equal or below 3 = Non-PI;<br>if equal or above 4 = PI<br>b) The biomedical career pathways listed were coded as described for PI Career Interest
Sum of Career Path Considered	Numerical	To what extent are you currently considering [the career path of] [INSERT CAREER PATHWAY TITLE]	1=Not at all considering<br>2=Slightly considering<br>3=Moderately considering<br>4=Strongly considering<br>5=Will definitely pursue	Sum of Bivariate Career Interests (0-20)<br><br>Each Career Path:<br>a) If equal or below 3 = less interested = 0<br>b) If equal or above 4 = highly interested = 1
The Familiarity with 20 Career Paths	NA	Which statement best describes your familiarity with the 20 career paths from the my Individual Development Plan (myIDP) shown in the Career Path Table	1=I am not familiar with any of these career paths<br>2=I am familiar with a few of these career paths (between 1 and 6)<br>3=I am familiar with some of these career paths (between 7 and 12)	

			4=I am familiar with most of these career paths (between 13 and 19)<br>5= I am familiar with all of these career paths	
Career Training Attained	N/A	I am getting the training I need for my desired career path(s)	0=Not applicable<br>1=Strongly disagree<br>2=Disagree<br>3=Neutral<br>4=Agree<br>5=Strongly agree	
Departmental Career Goal Support	Numerical	I am encouraged by my graduate program/department to pursue my career goals	0=Not applicable<br>1=Strongly disagree<br>2=Disagree<br>3=Neutral<br>4=Agree<br>5=Strongly agree	
PI Encouragement	Yes	I am encouraged by my PI/thesis advisor to pursue career development activities toward my career goals<br>I am encouraged by my PI/thesis advisor to pursue my career goals	0=I do not know<br>1=Strongly disagree<br>2=Disagree<br>3=Neutral<br>4=Agree<br>5=Strongly agree	
Citizenship	Yes	What is your citizenship status? (optional)	1=US citizen since birth<br>2=Naturalized US citizen<br>3=Non-US citizen with permanent resident visa (green card)<br>4=Non-US citizen with temporary US visa	US = United States of America<br><br>Citizen = 1 or 2<br>Non-Citizen = 3 or 4
Statistical Terms	N/A			ANOVA= analysis of variance<br>Tukey Corrections = for multiple comparisons
Other Terms	N/A			IRB=institutional review board<br>NIH BEST=National Institutes of Health Broadening Experiences in Scientific Training award<br>STEM=Science Technology Engineering and Math

S2 Table

Supplemental Table 2. Inter-reliability of Items (α = 0.86)

Deleted Variable	Item Total Correlation	Alpha (Item Deleted)
Item 1. Self-Assess abilities to pursue desired…	0.71	0.83
Item 2. Determine the steps to pursue desired…	0.74	0.82
Item 3. Seek advice from professionals in desired…	0.65	0.84
Item 4. Identify potential employers/institution…	0.66	0.84
Item 5. Achieve career goals	0.67	0.84

S3 Table 3. Full model ANOVA

Supplemental Table 3. 4-WAY ANOVA (Self-Efficacy with Gender x Citizenship x PI Career x Seniority)

ANALYSIS OF VARIANCE		
Variable(s)	F-test	p-value
**Citizenship**	**50.784**	**.000**
**Gender**	**42.873**	**.000**
PI Career	**302.833**	**.000**
Seniority	2.020	.155
**Citizenship x Gender**	**7.442**	**.006**
Citizenship x PI Career	.002	.968
Citizenship x Seniority	.858	.354
Gender x PI Career	.388	.533
Gender x Seniority	2.587	.108
PI Career x Seniority	.637	.425
Citizenship x Gender x PI Career	.083	.773
Citizenship x Gender x Seniority	1.639	.201
Citizenship x PI Career x Seniority	.114	.736
Gender x PI Career x Seniority	.255	.614
Citizenship x Gender x PI Career x Seniority	.085	.771

S4 Table 4. Contrasts (with Tukey corrections)

Tukey's multiple comparisons test	Significance	p-value
Male vs. Female	****	<0.0001
Male vs. Citizen Male Non-PI	****	<0.0001
Male vs. Citizen Male PI	****	<0.0001
Male vs. Citizen Female Non-PI	****	<0.0001
Male vs. Citizen Female PI	*	0.011
Male vs. Non-Citizen Male Non-PI	****	<0.0001
Male vs. Non-Citizen Male PI	*	0.014
Male vs. Non-Citizen Female Non-PI	****	<0.0001
Female vs. Citizen Male PI	****	<0.0001
Female vs. Citizen Female Non-PI	*	0.0299
Female vs. Citizen Female PI	****	<0.0001
Female vs. Non-Citizen Male PI	****	<0.0001
Female vs. Non-Citizen Female Non-PI	****	<0.0001
Citizen Male Non-PI vs. Citizen Male PI	****	<0.0001
Citizen Male Non-PI vs. Citizen Female PI	****	<0.0001
Citizen Male Non-PI vs. Non-Citizen Male PI	****	<0.0001
Citizen Male Non-PI vs. Non-Citizen Female Non-PI	****	<0.0001
Citizen Male PI vs. Citizen Female Non-PI	****	<0.0001
Citizen Male PI vs. Non-Citizen Male Non-PI	****	<0.0001
Citizen Male PI vs. Non-Citizen Female Non-PI	****	<0.0001
Citizen Male PI vs. Non-Citizen Female PI	****	<0.0001
Citizen Female Non-PI vs. Citizen Female PI	****	<0.0001
Citizen Female Non-PI vs. Non-Citizen Male PI	****	<0.0001

Citizen Female Non-PI vs. Non-Citizen Female Non-PI	****	<0.0001
Citizen Female Non-PI vs. Non-Citizen Female PI	**	0.005
Citizen Female PI vs. Non-Citizen Male Non-PI	****	<0.0001
Citizen Female PI vs. Non-Citizen Female Non-PI	****	<0.0001
Citizen Female PI vs. Non-Citizen Female PI	****	<0.0001
Non-Citizen Male Non-PI vs. Non-Citizen Male PI	****	<0.0001
Non-Citizen Male Non-PI vs. Non-Citizen Female Non-PI	*	0.033
Non-Citizen Male Non-PI vs. Non-Citizen Female PI	*	0.031
Non-Citizen Male PI vs. Non-Citizen Female Non-PI	****	<0.0001
Non-Citizen Male PI vs. Non-Citizen Female PI	****	<0.0001
Non-Citizen Female Non-PI vs. Non-Citizen Female PI	****	<0.0001

Supplemental Table 4 Legend. Tukey-corrected P-values for each significant contrast are listed exactly, accompanied by asterisks such that *p<.05, **p<.01, ***p<.001, ****p<.0001.

S5 Supplemental Figures

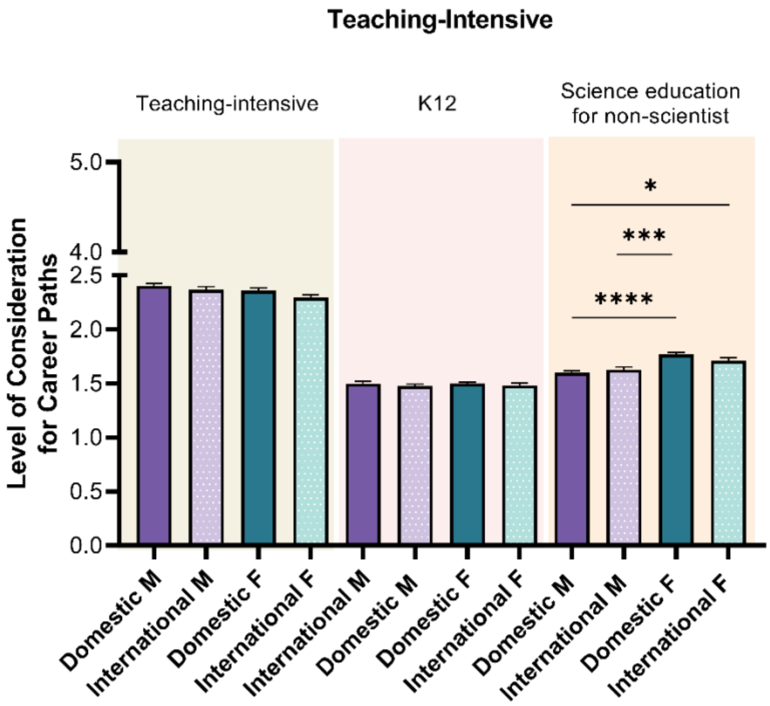

S5 Figure 1: Level of consideration for Teaching-Intensive career paths. Post-hoc t-tests were conducted between all possible pairings within each career path, as illustrated by each end of the respective bracket. P-values indicate significance of Tukey's multiple comparison tests, ****p<0.0001, ***p<0.001, and *p<0.05. *Note:* M=Male, F=Female. Color differences (purple and green) indicate the main variable gender, where purple = male, green = female; pattern indicates citizenship (no pattern = citizen; pattern = non-citizen).

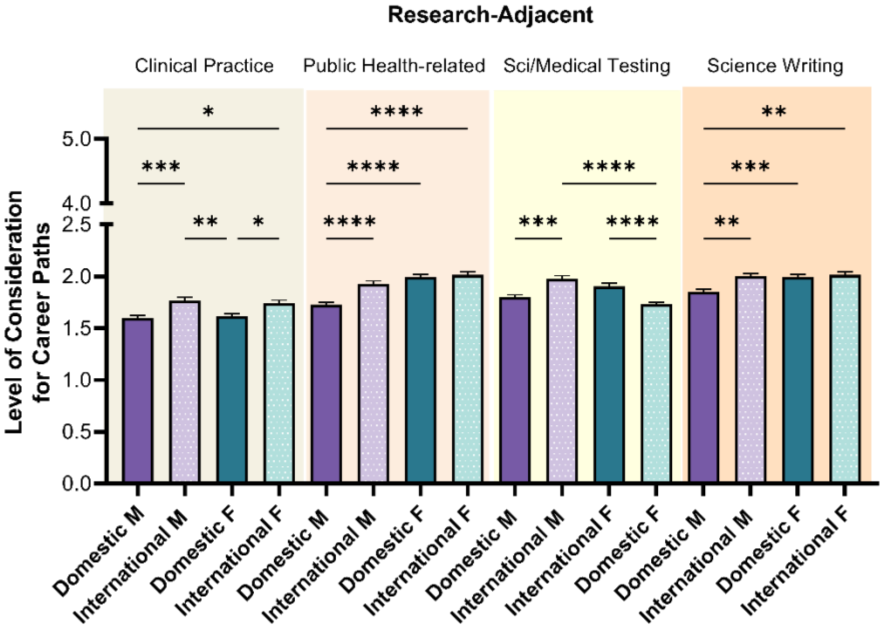

S5 Figure 2: Level of consideration for Research-Adjacent career paths. Post-hoc t-tests were conducted between all possible pairings within each career path, as illustrated by each end of the respective bracket. P-values indicate significance of Tukey's multiple comparison tests, ****p<0.0001, ***p<0.001, **p<0.01, and *p<0.05. *Note:* M=Male, F=Female. Color differences (purple and green) indicate the main variable gender, where purple = male, green = female; pattern indicates citizenship (no pattern = citizen; pattern = non-citizen).

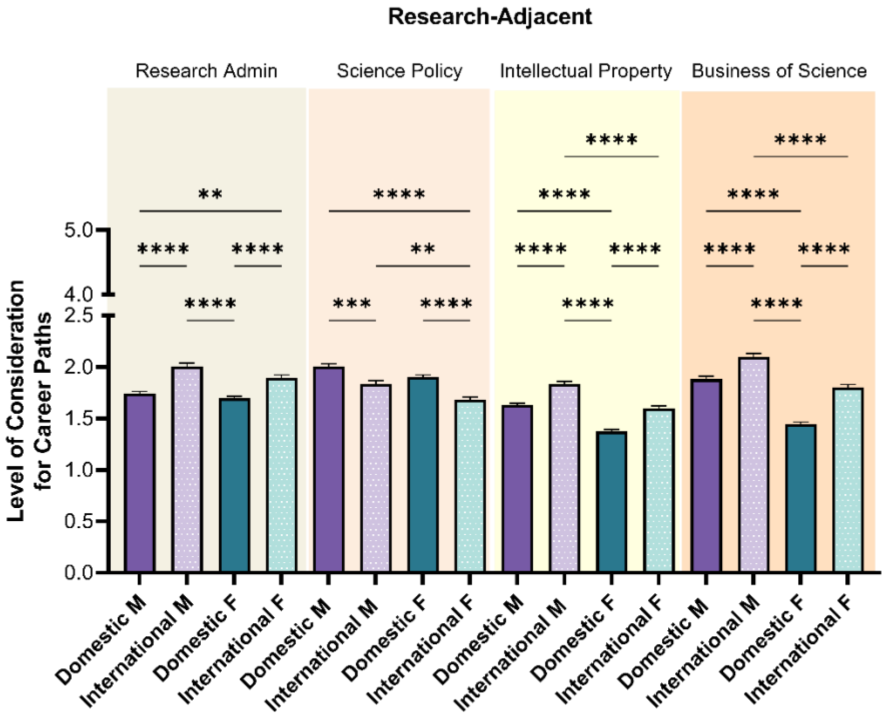

S5 Figure 3: Level of consideration for other Research-Adjacent career paths. Post-hoc t-tests were conducted between all possible pairings within each career path, as illustrated by each end of the respective bracket. P-values indicate significance of Tukey's multiple comparison tests, ****p<0.0001, ***p<0.001, and **p<0.01. *Note:* M=Male, F=Female. Color differences (purple and green) indicate the main variable gender, where purple = male, green = female; pattern indicates citizenship (no pattern = citizen; pattern = non-citizen).

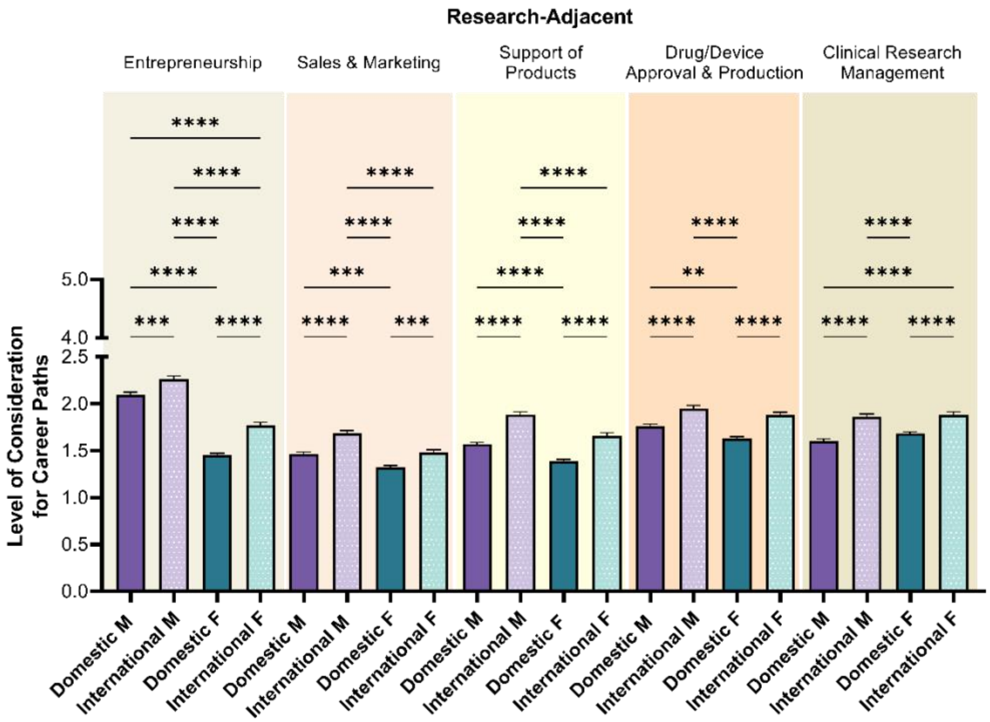

S5 Figure 4: Level of consideration for Research-Adjacent career paths. Post-hoc t-tests were conducted between all possible pairings within each career path, as illustrated by each end of the respective bracket. P-values indicate significance of Tukey's multiple comparison tests, ****p<0.0001, ***p<0.001, **p<0.01, and *p<0.05. *Note:* M=Male, F=Female. Color differences (purple and green) indicate the main variable gender, where purple = male, green = female; pattern indicates citizenship (no pattern = citizen; pattern = non-citizen).
